# The roles of BTG3 expression in gastric cancer: a potential marker for carcinogenesis and a target molecule for gene therapy

**DOI:** 10.18632/oncotarget.3734

**Published:** 2015-03-30

**Authors:** Wen-feng Gou, Xue-feng Yang, Dao-fu Shen, Shuang Zhao, Yun-peng Liu, Hong-zhi Sun, Yasuo Takano, Rong-jian Su, Jun-sheng Luo, Hua-chuan Zheng

**Affiliations:** ^1^ Cancer Research Center, Key Laboratory of Brain and Spinal Cord Injury of Liaoning Province, and Laboratory Animal Center, The First Affiliated Hospital of Liaoning Medical University, Jinzhou, China; ^2^ Department of Oncological Medicine, The First Affiliated Hospital of China Medical University, Shenyang, China; ^3^ School of Health Science, Tokyo University of Technology, Ohta-ku, Tokyo; ^4^ Experimental Center, Liaoning Medical University, Jinzhou, China

**Keywords:** gastric cancer, BTG3, carcinogenesis, pathobiological behaviors, gene therapy

## Abstract

*BTG* (B-cell translocation gene) can inhibit cell proliferation, metastasis and angiogenesis, cell cycle progression, and induce differentiation in various cells. Here, we found that BTG3 overexpression inhibited proliferation, induced S/G2 arrest, differentiation, autophagy, apoptosis, suppressed migration and invasion in MKN28 and MGC803 cells (*p* < 0.05). BTG3 transfectants showed a higher mRNA expression of p27, Bax, 14-3-3, Caspase-3, Caspase-9, Beclin 1, NF-κB, IL-1, -2, -4, -10 and -17, but a lower mRNA expression of p21, MMP-9 and VEGF than the control and mock (*p* < 0.05). At protein level, BTG3 overexpression increased the expression of CDK4, AIF, LC-3B, Beclin 1 and p38 (*p* < 0.05), but decreased the expression of p21 and β-catenin in both transfectants (*p* < 0.05). After treated with cisplatin, MG132, paclitaxel and SAHA, both BTG3 transfectants showed lower viability and higher apoptosis than the control in both time- and dose-dependent manners (*p* < 0.05). *BTG3* expression was restored after 5-aza-2′-deoxycytidine or MG132 treatment in gastric cancer cells. BTG3 expression was decreased in gastric cancer in comparison to the adjacent mucosa (*p* < 0.05), and positively correlated with venous invasion and dedifferentiation of cancer (*p* < 0.05). It was suggested that *BTG3* expression might contribute to gastric carcinogenesis. BTG3 overexpression might reverse the aggressive phenotypes and be employed as a potential target for gene therapy of gastric cancer.

## INTRODUCTION

Gastric cancer is still ranked as the fourth most common after cancers of the lung, breast, and colon and rectum since the second half of the 20th century. It is also the second most common cause of cancer-related death although advanced diagnostic and operative techniques are widely applied [1, 2]. It is helpful for the improvement of diagnosis, treatment and prevention to identify novel biomarkers and gene therapy targets during gastric carcinogenesis and subsequent progression.

BTG (B-cell translocation gene) family is composed of six proteins (BTG1, BTG2, BTG3, BTG4, Transducer of ErbB-2, and TOB2). The nucleocytoplamic translocation of BTG depends on cell growth states because both nuclear localization sequence and export signals exist in BTG3 protein [3, 4]. *BTG3* produces two mRNA transcripts due to 132-nucleotide deletion by alternative splicing [5-7]. In nucleus, BTG3 protein can bind to E2F1, Smad8 receptor-regulated Smad transcription factor, CCR4 transcription factor-associated protein Caf1 to finally suppress the proliferation and cell cycle progression [8-10]. Cheng et al. [11] found that BTG3 can be phosphorylated and activated *via* the interaction with checkpoint kinase 1 (CHK1). Additionally, BTG3 maintains genomic stability by promoting Lys63-linked ubiquitination and CHK1 activation. Lin et al. [12] demonstrated that BTG3 loss physiologically induced cellular senescence via ERK-AP1 signaling for acute induction of p16. Reportedly, BTG3 binds and suppresses Akt [13] and Ras/MAP kinase signaling [14] in cytoplasm.

BTG3-deficient mice might develop lung tumors by postnatal 21 months [15]. The down- regulated expression of BTG3 is documented in ovarian, lung, prostate or renal, hepatocellular cancer tissues or cells, and its expression is restored by the treatment with genistein and 5-aza-2′- deoxycytidine [6, 15-19]. Exogenous BTG3 overexpression inhibits the expression levels of matrix metalloproteinase (MMP)-2 and plasminogen activator inhibitor-1 in lung cancer cells [15]. Yanagida et al. [20] showed that BTG3 knockdown suppressed proliferation and tumorigenicity of ovarian clear cell carcinoma. Reportedly, BTG3 expression was inversely correlated with differentiation, distant metastasis and favorable prognosis of hepatocellular cancer (HCC). Ectopic BTG3 expression decreased proliferation, invasion and induced G_1_/S cycle arrest of HCC cells *in vitro* [18], and inhibited the growth of lung cancer *in vivo* [21]. To clarify the roles of *BTG3* in gastric carcinogenesis, we investigated the effects of BTG3 overexpression on cell proliferation, apoptosis, autophagy, senescence, invasion, migration and lamellipodia formation of gastric cancer cells and screened the expression of the phenotype- related genes. In addition, we examined the expression of *BTG*3 mRNA and protein in gastric cancer, non-cancerous mucosa and cancer cell lines, and compared them with clinicopathological parameters of cancer. Its promoter methylation was measured in gastric cancer cells and tissues. Finally, the *in vitro* and *in vivo* effects of BTG3 overexpression on aggressive behaviors of gastric cancer cells were determined in nude mice.

## RESULTS

### The expression and methylation of *BTG3* in gastric cancer cells

**Figure 1:**
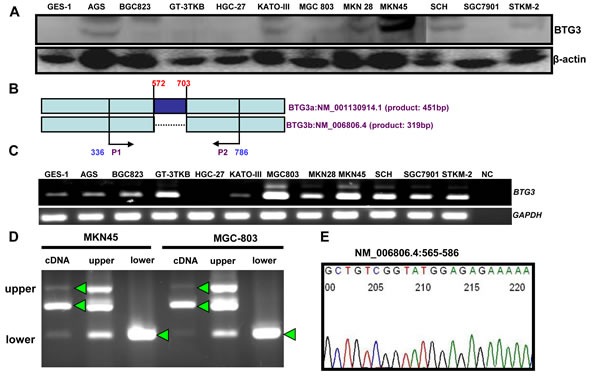

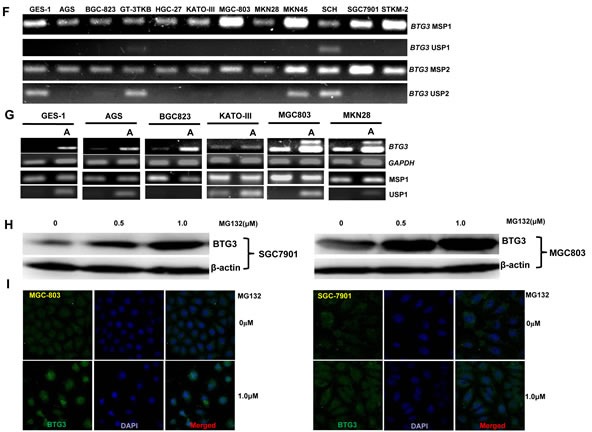
BTG3 expression and methylation in gastric cancer cell lines BTG3 protein expression (29 kDa) was detectable in gastric cancer cell lines at distinct levels with β-actin (42 kDa) as an internal control (**A**). We designed the BTG3 primers to determine its two different variants (**B**). Although three bands were observed for BTG3 amplicon, direct sequencing indicated that they are the same products (**C**-**E**). In addition, we found BTG3 methylation in gastric cancer or epithelial cell lines (**F**), but GES-1, AGS, BGC823, KATO-III, MGC803 and MKN28 cells showed a higher expression of *BTG*3 mRNA by RT-PCR after treated with 5-Aza-dC (**G**). MG 132 exposure increased BTG3 expression (**H** and **I**). NC, negative control; U, unmethylated; M, methylated; MSP, methylation-specific PCR; A, 5-Aza-dC; *, *p* < 0.05.

29kDa protein band of BTG3 was seen in AGS, BGC823, GT-3 TKB, HCG-27, KATO-III, MGC803, MKN28, MKN45, SCH, and STKM-2 (Figure 1A). To check *BTG3* mRNA expression, we designed the primers of RT-PCR targeting *BTG3*a (572–703, NM_001130914.1), which is deleted in *BTG3*b (Figure 1B). *BTG3* mRNA was detectable in gastric cancer and epithelial cells, except HGC-27 (Figure 1C). Three BTG3 bands were observed in MKN45 and MGC803 cells. The upper and lower DNA fragments were eluted and amplified (Figure 1D). All arrow-indicated bands proved to be *BTG3*b by direct DNA sequencing (Figure 1E). We detected the methylation of two promoter sites of *BTG3* in all cells by MSP, but unmethylation only in GES-1, GT-3 TKB, MKN45, and SCH (Figure 1F). *BTG3* expression was restored after the exposure to the demethylating agent 5-Aza-dC in GES-1, AGS, BGC823, KATO-III, MGC803 and MKN28 (Figure 1G, *p* < 0.05). The treatment of the proteasome inhibitor MG132 increased BTG3 expression in MGC803 and SGC7901 cells by Western blot (Figure 1H) and immunofluorescence (Figure 1I).

### The effects of BTG3 overexpression on the phenotypes and relevant molecules of gastric cancer cells

**Figure 2:**
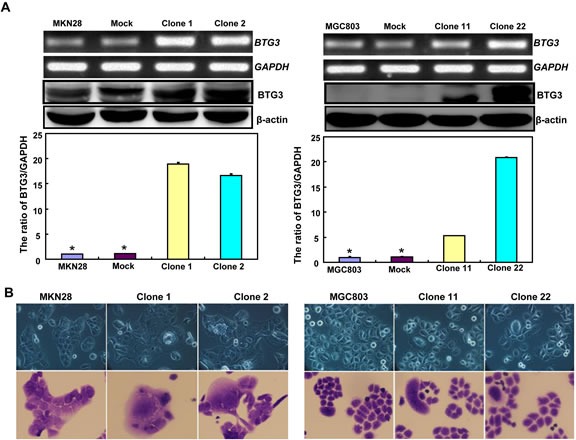

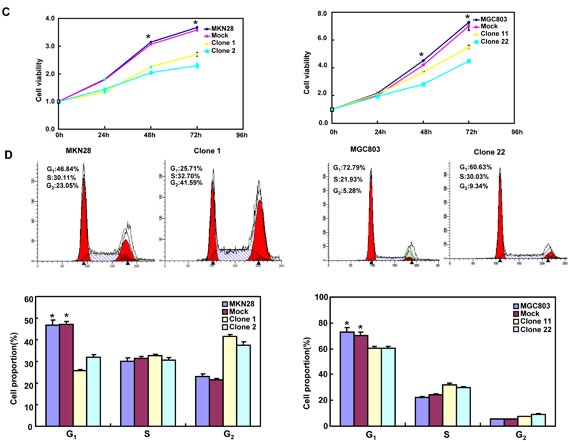

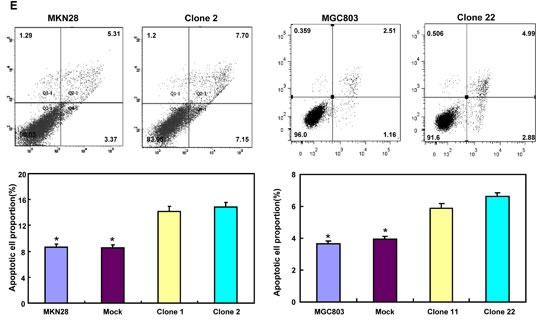

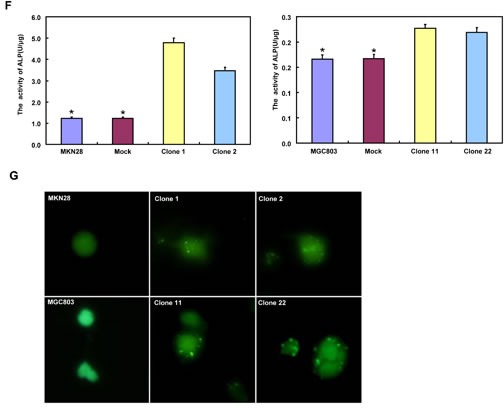

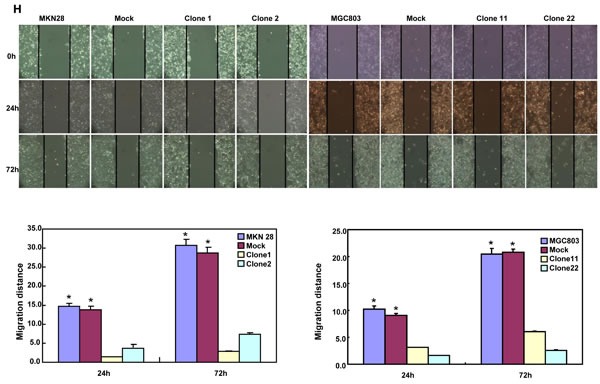

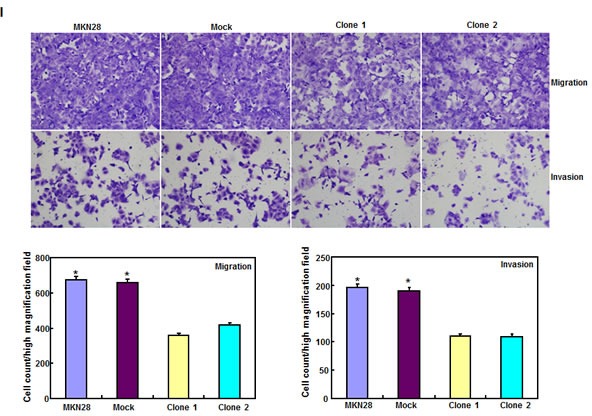

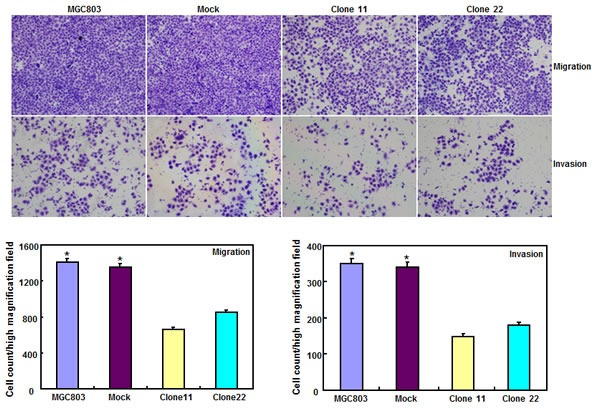

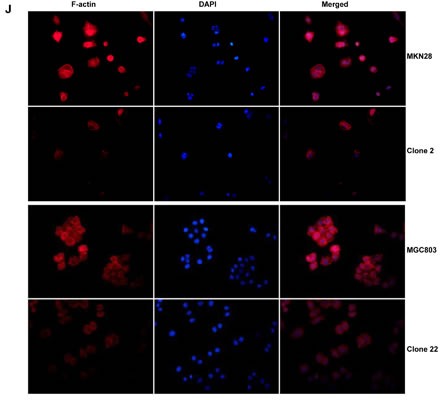

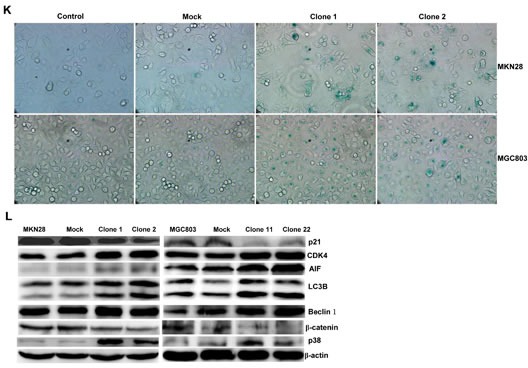

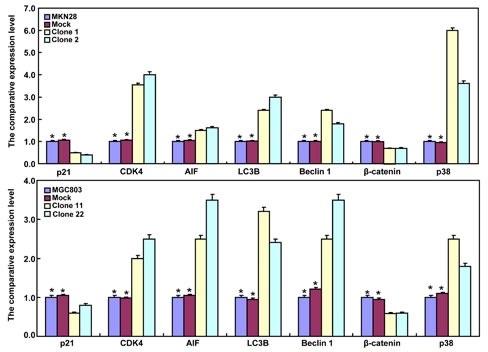

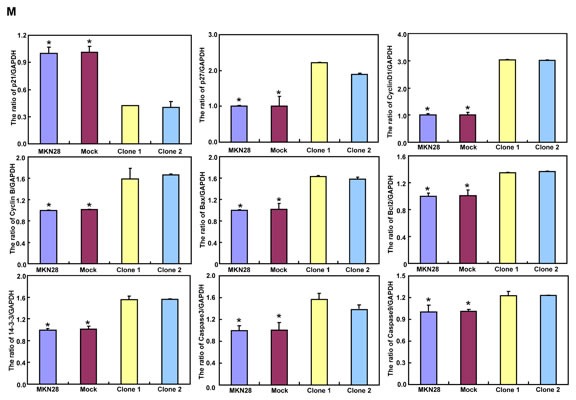

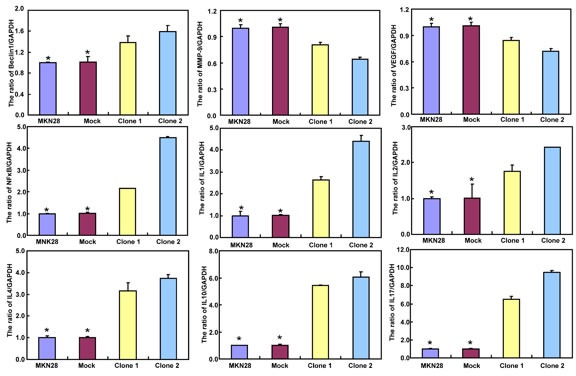

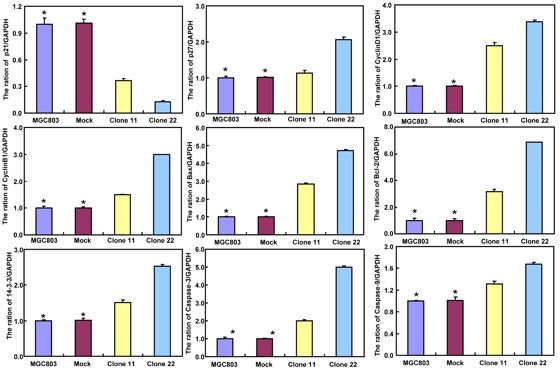

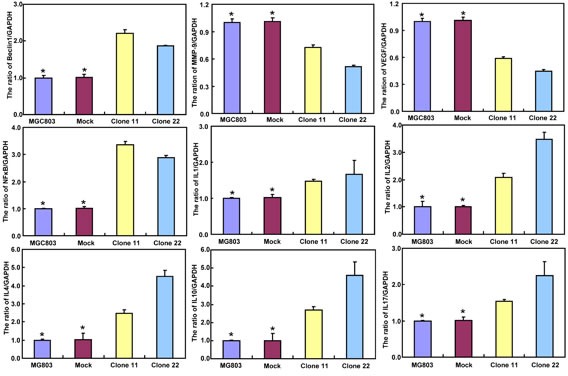
The effects of BTG3 overexpression on the phenotypes and expression of phenotype-related molecules in gastric cancer cells After transfection of pcDNA3.1-BTG3 into MKN28 and MGC803 cells, BTG3 expression became strong by RT-PCR and Western blot (**A**). BTG3 transfectants showed a high proportion of S-phase cells (**B**), slow growth (**C**), S/G_2_ arrest (**D**), apoptotic induction (**E**), better differentiation (**F**), high punctate LC3B-EGFP (**G**), weak ability to migrate and invade (**H**, **I**), weak lamellipodia formation (**J**) and high senescence (**K**) in comparison with control and mock. The expression of phenotype- related molecules was screened by Western blot (**L**) and real-time RT-PCR (**M**). *, *p* < 0.05, compared with thr transfectants.

BTG3-expressing plasmid was successfully transfected into MKN28 and MGC803 cells, evidenced by real-time PCR and Western blot (Figure 2A). Both transfectants showed a high disrupted mitosis (Figure 2B), a low growth (Figure 2C, *p* < 0.05) and S/G2 arrest (Figure 2D, *p* < 0.05) in comparison to the control and mock. There was a higher apoptotic level evidenced by Annexin V-FITC staining (Figure 2E, *p* < 0.05), a better differentiation by alkaline phosphatase (ALP) activity (Figure 2F, *p* < 0.05) and a higher autophagy by punctate LC3B-GFP accumulation (Figure 2G) and LC-3B expression (Figure 2L) in both BTG3 transfectants than the control and/or mock. According to the results of wound healing and transwell chamber assay, BTG3 transfectants also exhibited weaker ability to migrate and invade than the control and mock (Figure 2H and 2I, *p* < 0.05). Both transfectants showed weak F-actin staining (Figure 2J) and strong β-galactosidase staining in comparison to the control and/or mock (Figure 2K). Additionally, BTG3 transfectants showed a higher expression of *p27, Cyclin D1, Cyclin B1, Bax, Bcl-2, 14-3-3, Caspase-3, Caspase-9, Beclin 1, NF-κB, IL-1, IL-2, IL-4, IL-10* and *IL-17,* but a lower expression of *p21, MMP-9 and VEGF* than the control and mock (Figure 2M, *p* < 0.05). At the protein level (Figure 2L), BTG3 increased the expression of CDK4, AIF, LC-3B, Beclin 1, and p38 (*p* < 0.05), but decreased the expression of p21 and β-catenin in both MKN28 and MGC803 cells (*p* < 0.05).

**Figure 3:**
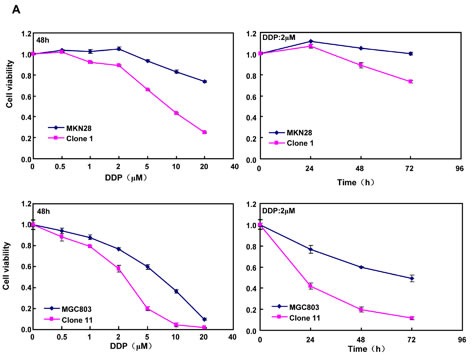

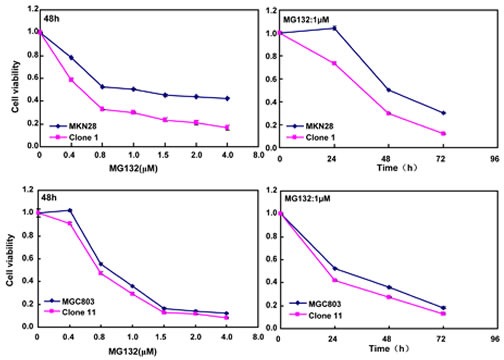

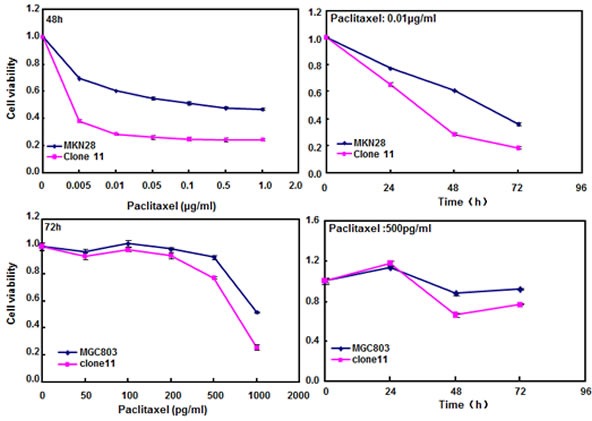

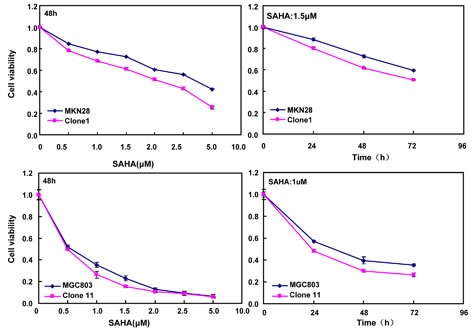

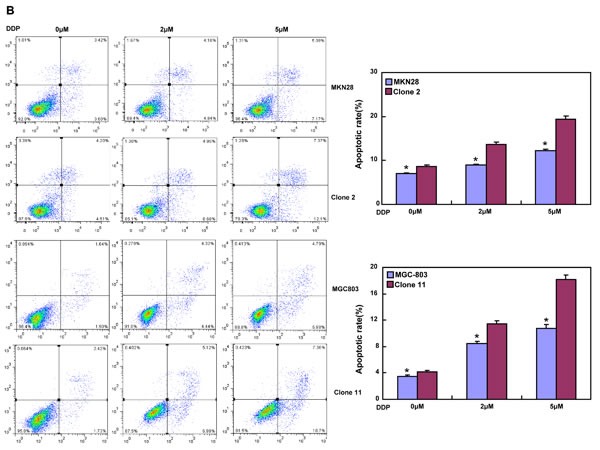

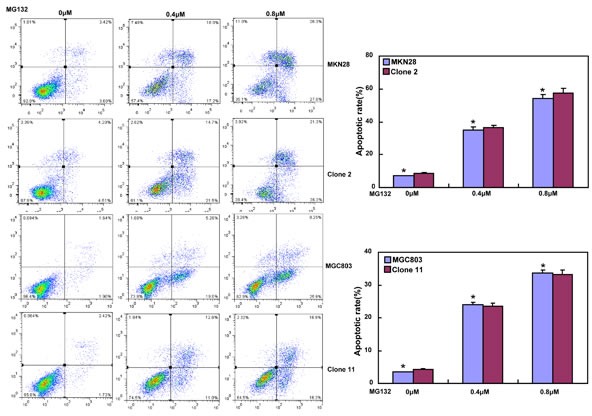

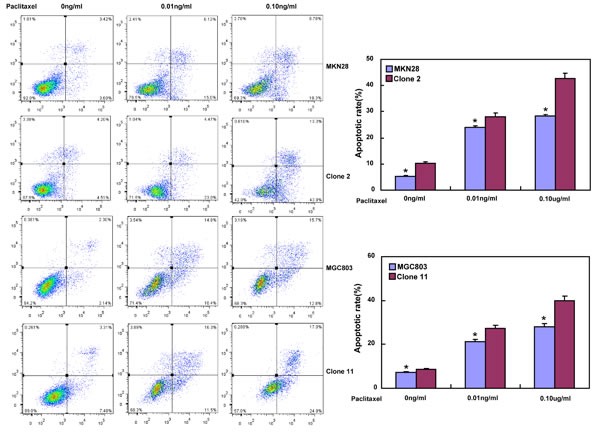

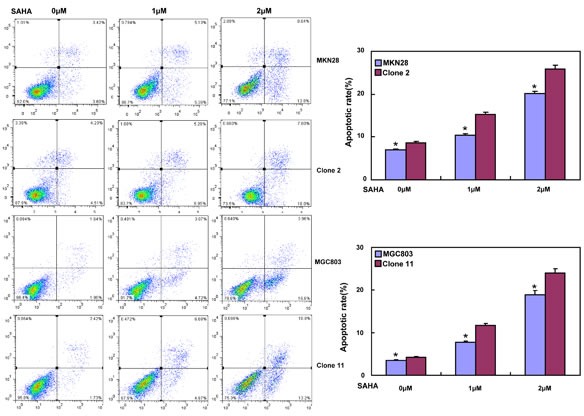

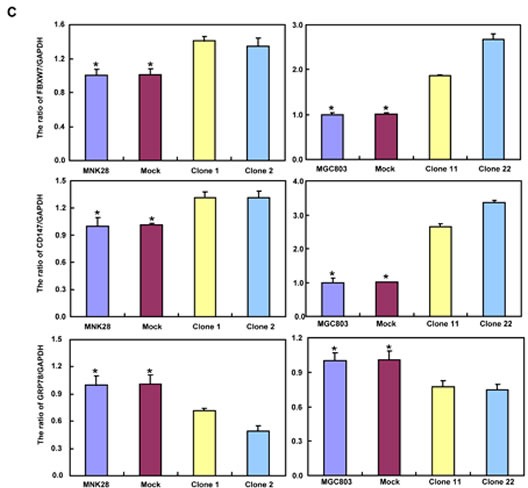

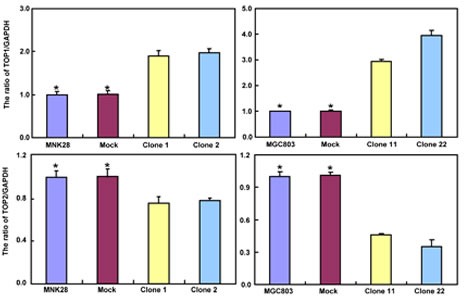
BTG3 overexpression enhances the sensitivity of gastric cancer cells to chemotherapeutic agents exposed to cisplatin (DDP), MG132, paclitaxel, and SAHA, BTG3 transfectants showed a lower viability and a higher apoptotic level than the control in both concentration and time-dependent manners (*p*<0.05, **A** and **B**). The chemoresistance-related genes were screened by real-time RT-PCR (**C**). * *p* < 0.05, compared with the transfectants.

After treated with different anti-cancer agents (cisplatin, MG132, paclitaxel and SAHA), both BTG3 transfectants showed lower viability and higher apoptosis than the control in both time- and dose-dependent manners (Figure 3A-3B, *p* < 0.05). BTG3 overexpression decreased the expression of *GRP78* and *TOP2,* but increased the expression of *FBXW7*, *CD147* and *TOP1* in both MKN28 and MGC803 cells (Figure 3C, *p* < 0.05).

### The expression and methylation of BTG3 in gastric cancer

**Figure 4:**
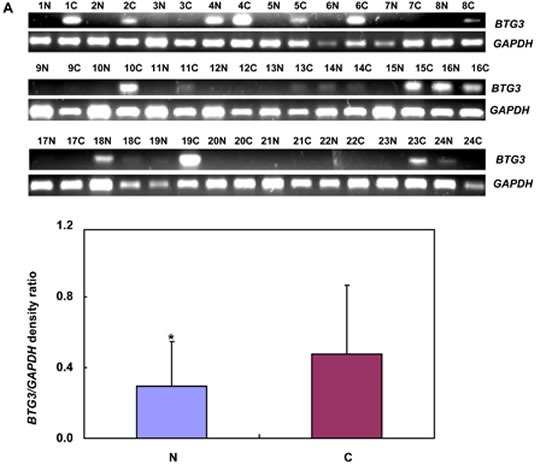

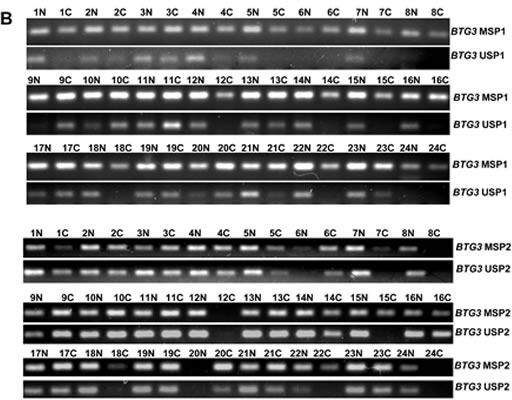

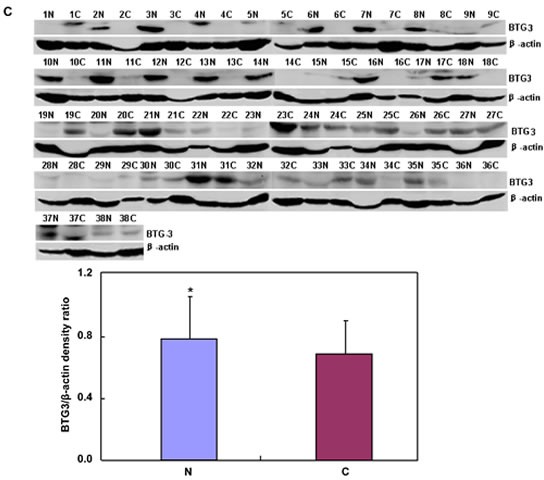
BTG3 expression and methylation in gastric cancer and matched mucosa *BTG3* cDNA was amplified by RT-PCR with *GAPDH* as an internal control. Densometric analysis showed higher BTG3 mRNA expression in carcinoma than paired mucosa (**A**). Methylation-specific PCR analysis was performed using primary tumors and matched mucosa tissues. The promoter methylation of BTG3 was detectable in all carcinoma or mucosa tissue (**B**). There was weaker BTG3 protein expression (29 kDa) in the tissue lysates of gastric cancer than matched mucosa with β-actin (42 kDa) as an internal control (**C**). Note: M, methylated; U, unmethylated; N, non-neoplastic mucosa; C, cancer; NC, negative control; *p* > 0.05.

Only *BTG3b* was amplified in 24 pairs of gastric cancer and corresponding mucosa. *BTG*3 mRNA expression was stronger in cancer than in the adjacent mucosa (Figure 4A, *p* < 0.05). To examine BTG3 methylation status, we designed methylation-specific primers of different *BTG3* promoter regions (−474-596 and −608-709) and found BTG3 methylation in all cases (Figure 4B). There was no correlation between the mRNA expression and promoter methylation of *BTG*3 (*p* > 0.05, data not shown). Among 38 cases of frozen gastric samples, 29kDa bands of BTG3 were weaker in gastric cancer than matched mucosa (Figure 4C, *p* < 0.05).

**Figure 5:**
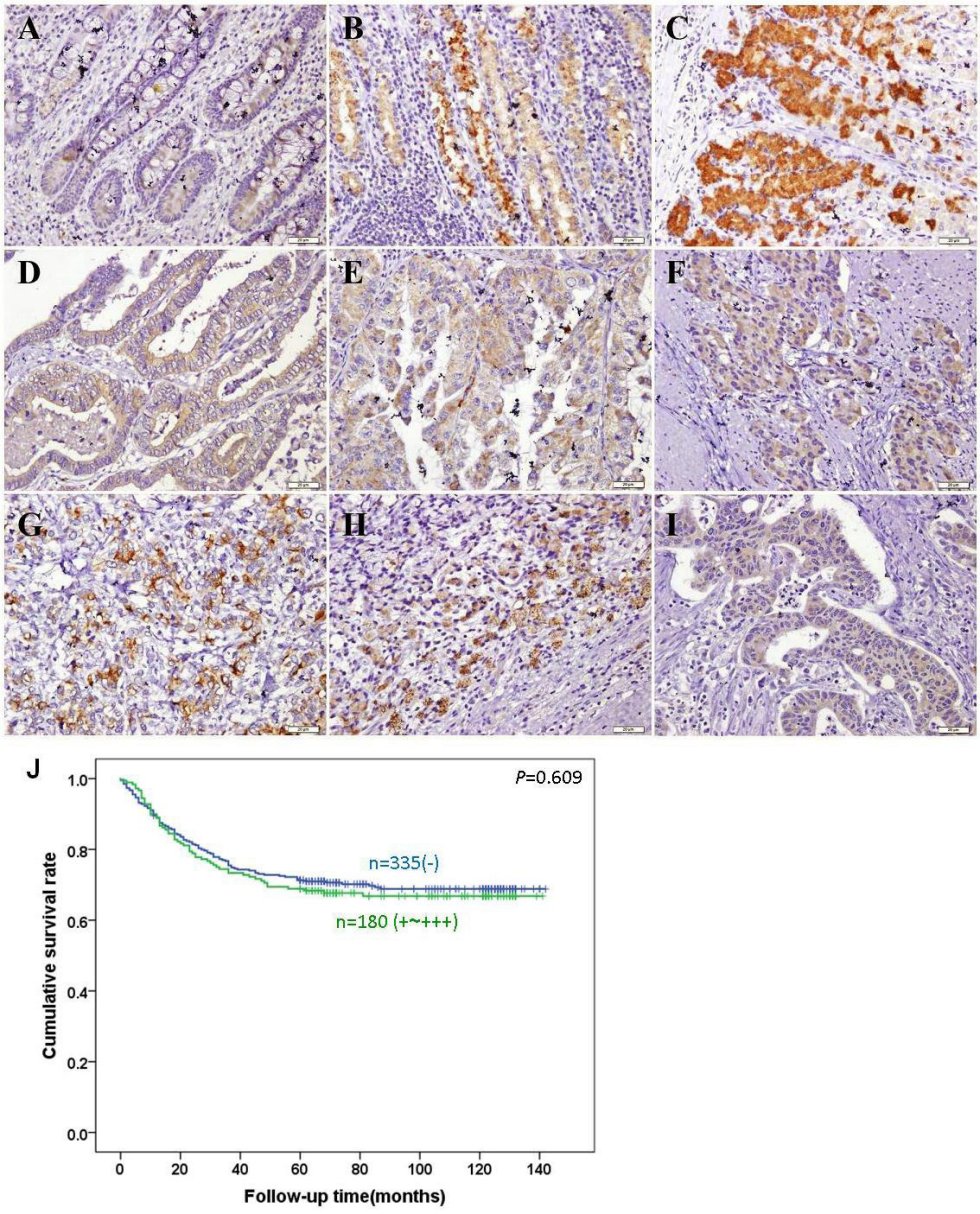
Immunohistochemical staining of BTG3 in gastric non-neoplastic mucosa and cancer BTG3 protein was positively detected in the cytoplasm of the superficial epithelial cells (**A**), deep propria glands (**B**, **C**), well- (**D**), moderately- (**E**), poorly- (**F**) differentiated, mucinous (**G**), signet ring cell (**H**) cancer and metastatic carcinoma in lymph node (**I**). Kaplan-Meier analysis showed no relationship between BTG3 protein expression and the cumulative survival rate of patients with gastric cancer (*p*>0.05, **J**).

As indicated in Figure 5, BTG3 expression was positively observed in the cytoplasm of superficial mucosa, deep propria glands, well-, moderately-, poorly-differentiated, mucinous, signet ring cell carcinoma and metastatic carcinoma in lymph node. BTG3 expression was detectable in gastric non-neoplastic mucosa (83.3%, 474/569), primary cancer (33.1%, 203/613), and metastatic cancer in lymph node (31.7%, 57/180), respectively. Statistically, BTG3 expression was decreased in gastric cancer in comparison to the adjacent mucosa (Table 1, *p* < 0.05).

**Table 1:** BTG3 expression in gastric non-neoplastic mucosa, primary and metastatic carcinomas

		BTG3 expression				
Groups	n	−	+	++	+++	PR(%)
Non-neoplastic mucosa	569	95	121	171	182	83.3
Primary carcinoma	613	410	134	49	20	33.1*
Metastatic carcinoma in lymph node	180	123	49	7	1	31.7

* compared with non-neoplastic mucosa, *p*<0.001.

PR=positive rate

### The correlation of BTG3 expression with clinicopathological parameters of gastric cancer

As summarized in Table 2, there was a positive correlation between BTG3 expression and venous invasion of gastric cancer (*p* < 0.05). In contrast, BTG3 positivity was not linked to age, sex, depth of invasion, lymphatic invasion, lymph node metastasis, distant metastasis or TNM staging of gastric cancer (*p* > 0.05). The diffuse-type carcinomas showed less BTG3 expression than intestinal- and mixed-type ones (*p* < 0.05), while no difference was observed in BTG3 expression between intestinal and diffuse components of mixed-type carcinomas (*p* > 0.05, data not shown). It was the same between primary and corresponding metastatic cancers (*p* > 0.05, data not shown).

**Table 2:** Relationship between BTG3 expression and clinicopathological features of gastric cancers

Clinicopathological features	BTG3 expression					
	n	−	+	++	+++	PR(%)	*p* value
Age(year)							0.426
<65	351	240	74	24	13	31.6	
≥65	262	170	60	25	7	35.1	
Sex							0.922
Male	427	284	93	36	14	33.5	
Female	186	126	41	13	6	32.3	
Depth of invasion							0.569
T_is-1_	294	203	56	20	15	31.0	
T_2-4_	310	201	75	29	5	35.2	
Lymphatic invasion							0.541
−	354	244	67	27	16	31.1	
+	259	166	67	22	4	35.9	
Venous invasion							0.004
−	346	251	63	18	14	27.5	
+	267	159	71	31	6	40.4	
Lymph node metastasis							0.679
−	348	232	72	29	15	33.3	
+	265	178	62	20	5	32.8	
Distant metastasis							0.268
−	578	385	125	48	20	33.4	
+	35	25	9	1	0	28.6	
TNM staging							0.145
I-II	355	248	65	26	16	30.1	
III-IV	244	152	65	23	4	37.7	
Lauren's classification							—
Intestinal-type	186	102	53	21	10	45.2	
Diffuse-type	219	177	28	9	5	19.2*	
Mixed-type	204	129	51	19	5	36.8	

PR=positive rate, T_is_=carcinoma in situ, T_1_=lamina propria and submucosa, T_2_=muscularis propria and subserosa, T_3_=exposure to serosa, T_4_=invasion into serosa, TNM= tumor-node-metastasis

* , compared with intestinal-type or mixed-type carcinomas, P<0.001

Follow-up information was available on 515 gastric cancer patients for periods ranging from 2 months to 10.8 years (median = 68.5 months). Univariate analysis using Kaplan-Meier indicated no significant difference between the cumulative survival rates of patients and BTG3 expression regardless of invasive depth (*p* > 0.05, Figure 5J). Multivariate analysis using Cox' s proportional hazard model indicated that lymph node metastasis, distant metastasis and tumor-node-metastasis(TNM) staging were independent prognostic factors for overall gastric cancers (Table 3, *p* < 0.05).

**Table 3:** Multivariate analysis of clinicopathological variables for survival with gastric carcinomas

Clinicopathological parameters	Relative risk (95%CI)	*p* value
Age(≥65years)	1.373(0.996-1.891)	0.053
Sex(female)	0.719(0.497-1.041)	0.081
Depth of invasion (T_2_-_4_)	2.088(0.689-6.329)	0.193
Lymphatic invasion(+)	1.501(0.955-2.359)	0.079
Venous invasion(+)	1.553(0.963-2.503)	0.071
Lymph node metastasis(+)	2.041(1.094-3.808)	0.025
Distant metastasis(+)	4.002(2.559-6.257)	<0.001
TNM staging(III-IV)	5.587(2.529-12.345)	<0.001
Lauren's classification(IT/DT/MT)	1.110(0.883-1.394)	0.372
BTG3 expression(+∼+++)	1.022(0.725-1.439)	0.903

CI=confidence interval, TNM= tumor-node-metastasis.

### BTG3 *in vivo* suppresses the growth of gastric cancer cells in nude mice

**Figure 6:**
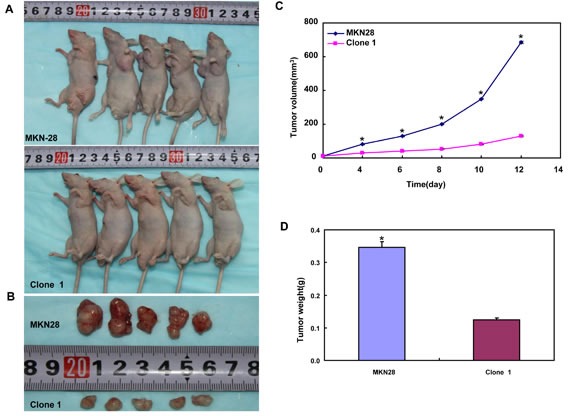

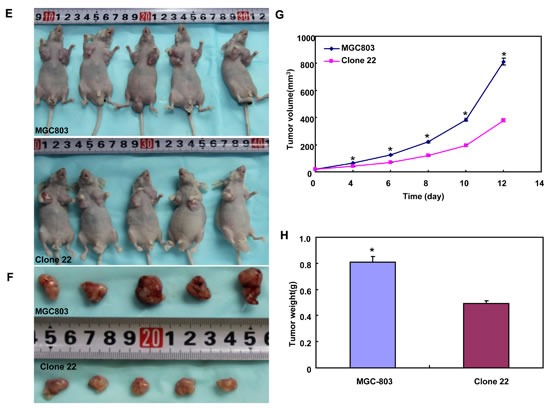

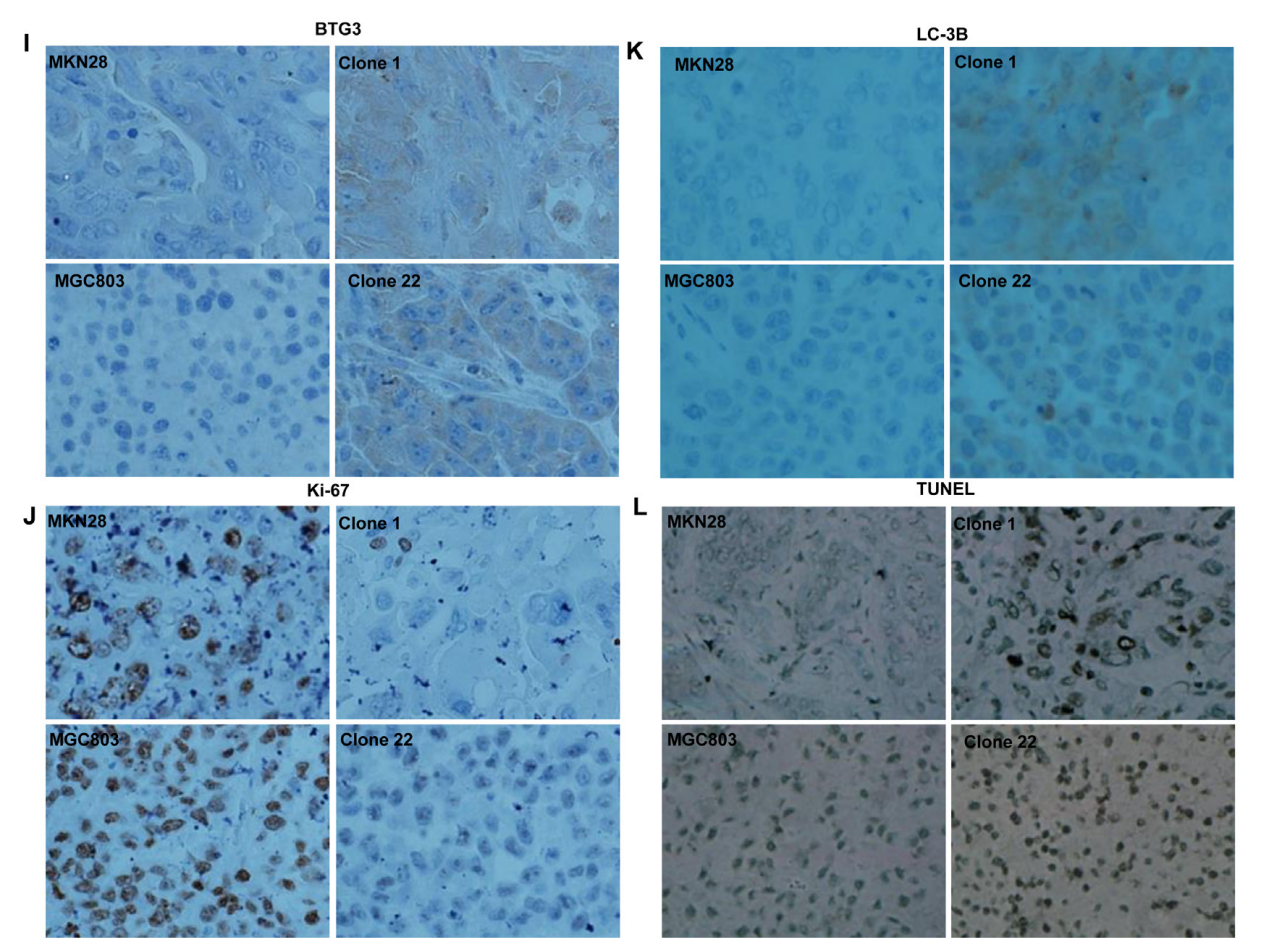
BTG3 suppresses the growth of gastric cancer cells in nude mice The tumor volume and weight of MKN28 (**A**-**D**) and MGC803 (**E**-**H**) cells were larger than their BTG3 transfectants. The transfectants showed stronger BTG3 (**I**) and LC-3B (**K**) expression, weaker ki-67 staining (**J**), and higher apoptotic signal by TUNEL (**L**) than the control.

MKN28, MGC803 and their BTG3 transfectants were subcutaneously transplanted into immune-deficient nude mice. The tumor volume and weight of both parental cells were larger and heavier than those of their BTG3 transfectants by ruling and weighting respectively (*p* < 0.05, Figure 6A-6H). Immunohistochemically both transfectants showed stronger BTG3 expression (Figure 6I), lower proliferation evidenced by ki-67 marker (Figure 5J) and more authophagy by LC-3B staining (Figure 6K) than the control. There appeared a higher apoptotic level in BTG3 transfectants than the control by TUNEL (Figure 6L).

## DISCUSSION

Here, we found that BTG3 protein was mainly localized in the cytoplasm of superficial mucosa, infiltrating inflammatory cells, deep propria glands, fundic glands, primary and metastatic cancers. Transcrptionally, *BTG*3b was only detectable in gastric cancer cells and tissues, while *BTG3*a and 3b in lung cancer cells and tissues [21]. Therefore, we concluded that alternative splicing and expression of *BTG3* had cell specificity. Based on the findings of immunohistochemistry and Western blot, BTG3 expression was reduced in gastric cancer, compared with adjacent mucosa, indicating that down- regulated BTG3 expression enhanced the malignant transformation of gastric epithelial cells. In contrast, it was the converse for *BTG3*b mRNA, opposite to other reports [19, 21, 22]. MG132- induced BTG3 overexpression indicated that BTG3 hypoexpression might be due to its ubiquitin- proteasome degradation in gastric cancer [23]. We treated cells with 5-Aza and observed the restoration of *BTG3* mRNA expression in gastric cancer cells, in line with the reports about breast, renal and prostate cancer cells [6, 16, 17]. However, no correlation was seen between *BTG3* methylation and mRNA expression in gastric cancer and paired mucosa. Taken together, it was suggested that *BTG3* methylation was partially responsible for its silenced expression.

Reportedly, BTG3 expression was negatively correlated with lymph node metastasis of lung cancer [21], distant metastasis of gastric [22] and hepatocellular [18] cancers. Provenzani et al. [24] found that *BTG3* mRNA had about 1.83 fold higher in colon primary than metastatic carcinoma according to cDNA microarray. Here, we found that BTG3 expression was positively linked to venous invasion. BTG3 overexpression *in vitro* decreased migration, invasion and lamellipodia formation of gastric cancer cells with a down-regulated expression of MMP-9 and VEGF. These findings suggested that BTG3 overexpression might inhibit invasion and metastasis by reducing MMP-9 and VEGF expression. Additionally, a higher BTG3 expression was found in intestinal- than diffuse-type carcinoma, in line with a previous report [22]. Therefore, we hypothesize that differential BTG3 expression underlies the molecular mechanisms of both gastric carcinomas, which is also supported by ALP results. Mixed-type carcinoma showed BTG3 expression between the other two subtypes and no difference in BTG3 expression in its intestinal and diffuse components, supporting the opinion that different components of mixed-type carcinoma might originate from common stem cells [25].

In the present study, BTG3 overexpression *in vitro* inhibited proliferation, induced apoptosis and senescence, and S/G_2_ arrest of gastric cancer cells, and *in vivo* suppressed the growth of gastric cancer cells by inhibiting proliferation, inducing apoptosis and autophagy. These data indicate that BTG3 might be employed as a molecular target of gene therapy to reverse the aggressive phenotypes of gastric cancer. Reportedly, both Cyclin D and E activate CDKs and are involved in G_1_-S transition, which is inhibited by p21 and p27. 14-3-3 sequesters Cdc25C to the cytoplasm to prevent the interactions with Cyclin B-Cdk1 necessary for G_2_/M transition [26]. BTG3-mediated S/G_2_ arrest was positively linked to 14-3-3 overexpression and p21 hypoexpression regardless of CDK4 overexpression. The apoptosis-inducing effect of BTG3 might result from the overexpression of Bax, AIF, Caspase-3 and -9 because AIF, Caspase-3 and -9 initiate apoptosis, and Bax opens the mitochondrial voltage-dependent anion channel for apoptosis [27]. Here, BTG3 overexpression was found to *in vivo* and *vitro* induce the autophagy of gastric cancer cells with Beclin 1 overexpression, indicating that BTG3-mediated autophagy was dependent on beclin-1 expression [28]. Moreover, we demonstrated that BTG3 enhanced the sensitivity of gastric cancer cells to chemotherapeutic agents, which was positively associated with their apoptotic induction and the down-regulated expression of drug resistance genes (GRP78 and TOP2) [29, 30]. In the present study, we also found that BTG3 overexpression might ameliorate β-catenin, promote p38 and NF-κB expression in gastric cancer cells, indicating that BTG3 might target these signal pathways.

Although Ren et al. [22] reported that BTG3 expression was positively associated with favorable prognosis of gastric cancer, the BTG3-positive case number is too small (n = 8). In our group, BTG3 hypoexpression was found to correlate with a short disease-free time and overall survival of ovarian cancer patients [19]. However, no link between BTG3 expression and the survival of 515 gastric cancer patients was revealed even though stratified by depth of invasion. Multivariate analysis demonstrated that lymph node metastasis, distant metastasis and TNM staging were independent prognostic factors for overall gastric cancer. These findings suggested that BTG3 expression could not be employed to indicate the prognosis of gastric cancer patients.

In conclusion, down-regulated BTG3 expression might have impact on the malignant transformation of gastric epithelial cells and should be considered as a good biomarker for gastric carcinogenesis. Promoter methylation of *BTG*3 is partially responsible for its down-regulated expression. BTG3 overexpression might reverse the aggressive phenotypes of gastric cancer cells and be employed as a target molecule for gene therapy.

## MATERIALS AND METHODS

### Cell culture

Gastric cancer cell lines, MKN28, AGS, BGC823, MGC803, MNK45 and SGC7901, KATO-III, HGC-27, GT-3TKB and STKM-2, SCH and gastric epithelial cell line, GES-1 come from Japanese Physical and Chemical Institute, Tokyo, Japan, Beijing Institute for Cancer Research, Beijing, China, and Cell bank of Chinese Academy of Sciences, Shanghai, China, respectively. They were maintained in RPMI 1640, MEM, DMEM or Ham F12 medium supplemented with 10% fetal bovine serum (FBS), 100 units/mL penicillin, and 100 μg/mL streptomycin in a humidified atmosphere of 5% CO_2_ at 37°C. To demethylate the genomic DNA, cells were treated with 5 mmol/L of 5-aza-2′- deoxycytidine (5-Aza-dC, DNA demethylating agent) for 72 h and then harvested to extract DNA and RNA. To check the drug sensitivity, we exposed cells to cisplatin (a platinum-containing DNA crosslinker), MG132 (a proteasome inhibitor), paclitaxel (a mitotic inhibitor), and SAHA (a histone deacetylase inhibitor).

### Plasmid construction and transfection

*BTG*3 gene was amplified using sense: 5′-ATGAAGAATGAAATTGCTGCCGTTG-3′, antisense: 5′-GTGAGGTGCTAACATGTGAGGATT-3′ and the template cDNA from MKN28. The PCR products was phosphorylated by BKL kit and ligated with *EcoR*V-digested and dephosphorylated pcDNA3.1. MKN28 and MGC803 cells were transfected with pcDNA3.1-BTG3, pcDNA3.1 vector or pEGFP-N1-LC-3B at 24 h after seeding on dishes, and selected by G418 with monoclone collection.

### Proliferation assay

Cell counting Kit-8 (CCK-8, Japan) was employed to determine the number of viable cells. In brief, 2.5 × 10^3^cells/well were seeded on 96-well plate and allowed to adhere. At different time points, 10 μL of CCK-8 solution was added into each well of the plate and the plates were incubated for 3 h in the incubator and measured at 450 nm.

### Cell cycle analysis

The cells were trypsinized, collected, washed by PBS twice and fixed in cold 10mL ethanol for more than 2 h. Then, the cells were washed by PBS twice and incubated with 1mL RNase (0.25 mg/mL) at 37°C for 1 h. The cells were pelleted and resuspended in propidium iodide (PI) at a concentration of 50 μg/mL and incubated at 4°C in the dark for 30 min. Finally, flow cytometry was employed to examine PI signal.

### Apoptosis assay by flow cytometry

Flow cytometry was performed with 7-amino-actinomycin (7-AAD) and FITC-labeled Annexin V (BD Pharmingen, San Diego, CA92121) to detect phosphatidylserine externalization as an endpoint indicator of apoptosis according to the manufacturer's instructions.

### Wound healing assay

Cells were seeded at a density of 1.0 × 10^6^ cells/well in 6-well culture plates. After they had grown to confluence, the cell monolayer was scraped with a pipette tip to create a scratch, washed by PBS for three times and cultured in the FBS-free medium. Cells were photographed at 24 h, and 72 h with the scratch area measured using Image software.

### Transwell chamber assays

For invasive assay, 2.5 × 10^5^ cells were resuspended in serum-free RPMI 1640, and seeded in the matrigel-coated insert on the top portion of the chamber (BD Bioscience, 354481). The lower compartment of the chamber contained 10% FBS as a chemoattractant. After incubated at 37°C and 5% CO_2_ for 24 h, cells on the membrane were scrubbed, washed with PBS, fixed in 100% methanol and stained with Giemsa dye. For migration assay, the procedures were the same as above excluding membrane-control insert (BD Bioscience).

### Alkaline phosphatase (ALP) activity

ALP activity was used as a marker of colorectal differentiation. The cells were harvested, broken and subjected to the determination of ALP activity using Diagnostics ALP reagent (Sigma, USA). The protein content of the samples was determined Biorad protein assay kit (Biorad, USA). ALP activity was calculated as U per μg of protein.

### β-galactosidase staining

β-galactosidase staining was performed with a senescence-associated β-galactosidase staining kit (Beyotime, Shanghai, China). Cells were washed three times with PBS and fixed with 4% paraformaldehyde for 15 min at room temperature. Next, cells were incubated overnight at 37°C in the dark with the working solution containing 0.05 mg/mL X-gal. Finally, cells were examined under a light inverted microscope (Olymphus).

### Immunofluorescence

Cells were grown on glass coverslips, fixed with 4% formaldehyde for 10 min, and permeabilized with 0.2% Triton X-100 for 10 min at room temperature. After washed with PBS, cells were incubated overnight at 4°C with anti-BTG3 (Sigma, USA) or -LC-3B(Cell Signaling, USA). The Alexa Fluor^®^ 488 IgG (Invitrogen) was used as secondary antibody. Alexa Fluor^®^ 568 phalloidin (invitrogen) was employed to observe the lamellipodia. Nuclei were stained with 1 μg/ml DAPI (Sigma) at 37°C. Finally, coverslips were mounted with SlowFade^®^ Gold antifade reagent (Invitrogen) and observed under a laser confocal microscope (Olympus).

### Subjects and pathology

Gastric adenocarcinomas (*n* = 613), adjacent non-neoplastic mucosa (*n* = 569) and lymph node with metastases (*n* = 180) were collected from surgical resection in the Affiliated Hospital of Kanagawa Cancer Center (Japan). The patients with gastric cancer were 427 men and 186 women (24–87 years, mean = 62.1years). Among them, 265 cases have tumors accompanied with lymph node metastasis, 35 with distant metastasis. Intestinal and diffuse components of 114 mixed-type carcinomas were punched out for tissue microarray. Fresh gastric cancer and adjacent non-neoplastic mucosa were collected from our hospital and frozen in −80°C until DNA, RNA and protein extraction. None of the patients underwent chemotherapy, radiotherapy or adjuvant treatment before surgery. They or their relatives provided written consent for use of tumor tissue for clinical research. The Ethical Committee of our university and Kanagawa Cancer Center approved the research protocol. We followed up the patients by consulting their case documents and through telephone.

All tissues were fixed in 10% neutral formalin, embedded in paraffin and sections were cut at 4 μm. These sections were stained by hematoxylin-and-eosin (HE) to confirm their histological diagnosis and other microscopic characteristics. The tumor-node-metastasis staging for gastric cancer was evaluated according to the Union Internationale Contre le Cancer system [31]. Histological architecture of gastric carcinoma was expressed in terms of Lauren's classification [25]. Furthermore, tumor size, depth of invasion, lymphatic and venous invasion were determined.

### RT-PCR and direct sequencing

Total RNA was extracted from gastric carcinoma and epithelial cell lines or cancer tissues using QIAGEN RNeasy mini kit (Germany). Two micrograms of total RNA was subjected to cDNA synthesis using AMV transcriptase and random primers (Takara, Japan). According to the Genbank, oligonucleotide primers for PCR were shown Supplementary Table 1. General and real-time RT-PCR amplification was performed using Hotstart Taq polymerase (Takara) and SYBR Premix Ex Taq^TM^ II kit (Takara) respectively. The amplicons were electrophoresed in 2% agarose gel for 30 min and densitometric quantification was performed by comparison to *GAPDH* using Scion Image software. PCR products were purified with QIAquick gel extraction kit (QIAGEN) and then sequenced using a BigDye Terminator v 3.1 cycle sequencing kit (Applied Biosystems). The sequence data was compared with the human *BTG3* cDNA using BLAST.

### Methylation-specific PCR (MSP)

Genomic DNA was extracted from cells and tissues using QIAamp DNA Mini kit (QIAGEN). DNA was modified chemically with sodium metabisulphite. The bisulfite-modified DNA was amplified by using primer pairs that specifically amplify either methylated or unmethylated *BTG3* as described previously [32]. The following methylated *BTG3*-specific primers were used: sense: 5′-TAAAATATAGTAGGGCGGTTGTACG-3′; and antisense: 5′AAACTTCATAAAACACGAACTC G-3′ (*BTG3* MSP1: −474-596) or sense: 5′-AGTTGGGTTTAGAATCGTTATTC-3′ and anti-sense: 5′-ACTTAATCCTTTCGACTATCTCGAC-3′ (*BTG3* MSP2: −608-709). The corresponding unmethylated primers ware sense: 5′-TAAAATATAGTAGGGTGGTTGTATGG-3′ and antisense: 5′-AAACTTCATAAAAAACACAAACTCAAC-3′ (*BTG3* USP1: −474-596) or sense: 5′-GAGTTG GGTTTAGAATTTGTTATTTTG-3′ and anti-sense: 5′-ACTTAATCCTTTCAACTATCTCAAC-3′ (*BTG3* USP2: −608-709). MSP was performed for 40 cycles using hot-start polymerase (Takara).

### Western blot

The cancer tissues and cells were subjected to protein extraction by homogenization or sonication in RIPA lysis buffer. Denatured protein was separated on SDS-polyacrylamide gel and transferred to Hybond membrane, which was then blocked overnight in 5% skim milk in TBST. For immunoblot, the membrane was incubated for 15 min with the primary antibody (Supplementary Table 2). Then, it was rinsed by TBST and incubated with IgG conjugated to horseradish peroxidase (DAKO) for 15 min. All the incubations were performed in a microwave oven to allow intermittent irradiation as recommended by Li et al. [33]. Bands were visualized with X film by ECL-Plus detection reagents (Santa Cruz, USA). Densitometric quantification of target proteins was performed with β-actin control using Scion Image software.

### Xenograft models

Locally bred female BALB/c nude (nu/nu) mice, 6–8 weeks of age at implantation, were used. The animals were maintained under specific pathogen-free conditions, and food and water were supplied ad libitum. Housing and all procedures involving animals were performed according to protocols approved by the Committee for Animal Experiments guidelines on animal welfare of Liaoning Medical University. Subcutaneous xenografts were established by injection of 1× 10^6^ tumor cells per mouse to the axilla (*n* = 10 / group). Until the end of the experiment, one mouse from each group was randomly selected to be anesthetized, photographed, and sacrificed. For each tumor, measurements were made using calipers, and tumor volumes were calculated as follows: length × width × depth × 0.52. The part of tumors were subsequently fixed in 4% paraformaldehyde for 24 h, and then embedded in paraffin.

### Tissue microarray and immunohistochemistry

Under the guaidance of HE-stained slides, a two mm-in-diameter tissue core per donor block was punched out and transferred to a recipient block with a maximum of 48 cores using a Tissue Microarrayer (Japan). Consecutive sections were deparaffinised with xylene, rehydrated with alcohol, and subjected to antigen retrieval by irradiating in target retrieval solution (DAKO) for 15 min with microwave oven (Oriental rotor Lmt. Co., Tokyo, Japan). All procedures were performed as described previously [34]. Omission of the primary antibody was used as a negative control.

As indicated in Figure 3, BTG3 protein was positively localized in the cytoplasm. One hundred cells were randomly selected and counted from 5 representative fields of each section blindly by two independent observers. The inconsistent data were confirmed by both persons until final agreements were reached. The expression was graded and counted as follows: 0 = negative; 1 = 1–50%; 2 = 50–74%; 3 ≥75%. The staining intensity score was graded as follows: 1 = weak; 2 = intermediate; and 3 = strong. The scores for BTG3 positivity and staining intensity were multiplied to obtain a final score, which determines their expression (– = 0; + = 1–2; ++ = 3–5; +++ = 6–9).

### Terminal digoxigenin-labeled dUTP nick-end labeling (TUNEL)

Cell apoptosis was assessed using TUENL, a method that is based on the specific binding O-TdT to the 3-OH ends of DNA, ensuring the synthesis of a polydeoxynucleotide polymer. For this purpose, ApopTag Plus Peroxidase In Situ Apoptosis Detection Kit (Chemicon) was employed according to the recommendation. Omission of the working strength TdT enzyme was considered as a negative control.

### Statistical analysis

Statistical evaluation was performed using *Spearman's* correlation test to analyze the rank data and *Mann-Whitney* U to differentiate the means of different groups. *Kaplan-Meier* survival plots were generated and comparisons between survival curves were made with the log-rank statistics. *Cox*'s proportional hazards model was employed for multivariate analysis. SPSS 10.0 software was applied to analyze all data and *p* < 0.05 was considered statistically significant.

## SUPPLEMENTARY TABLES


