# Supplementary material for: The roles of BTG3 expression in gastric cancer: a potential marker for carcinogenesis and a target molecule for gene therapy
Source: Oncotarget. 2015 Mar 30;6(23):19841–67. doi: 10.18632/oncotarget.3734 (PMC4637325; doi:10.18632/oncotarget.3734)
Supplement: Supplementary file 1 [file oncotarget-06-19841-s001.pdf]

The roles of BTG3 expression in gastric cancer: a potential marker for carcinogenesis and a target molecule for gene therapy

Supplementary Material

Supplementary Table 1 The primers used in RT-PCR

	Names	Primer' s sequence	Distribution	AT(°C)	Product size(bp)	Extension time(s)
1	*BTG3*	F: 5'-GCAGTTGAGAGGTTTGCTGA-3'<br>R: 5'-TAACTTTCCTGGAGATCTCATT- 3'	NM_ 01130914.1<br>NM_006806.4	60	451/319	34
2	*P21*	F: 5'-ACTGTCTTGTACCCTTGTGCC-3'<br>R: 5'-AAATCTGTCATGCTGGTCTGC-3'	XM_003950827<br>572-679	60	108	34
3	*P27*	F: 5'-GGCTCCGGCTAACTCTGA-3'<br>R: 5'-TTCTTCTGTTCTGTTGGCTCTT-3'	XM_522347<br>1081-1237	60	157	34
4	*CyclinD1*	F: 5'-TGCCACAGATGTGAAGTTCATT-3'<br>R: 5'-CAGTCCGGGTCACACTTGAT-3'	NG_000002<br>776-937	60	162	34
5	*Cyclin B1*	F: 5' -GTTATGCAGCACCTG-3'<br>R: 5'-CTTGGCTAAATCTTGAACT-3'	NM_001088590<br>1388-1537	60	150	34
6	*Bax*	F: 5'-GATTGCCGCCGTGGAC-3'<br>R: 5'-GCCCCAGTTGAAGTTGC-3'	DQ926869<br>306-393	60	88	34
7	*Bcl-2*	F: 5'-GCCTTCTTTGAGTTCGGTGGG-3'<br>R: 5'-TGTGCAGGTGCCGGTTCAG-3'	DQ926871<br>938-1052	60	115	34
8	*14-3-3*	F: 5'-CAAAGACAGCACCCTCA-3'<br>R: 5'-TTCTGCCGCATCACAT-3'	XM_010379682.1<br>845-935	60	91	34
9	*Casepase3*	F: 5'-CAGAACTGGACTGTGGCATTG-3'<br>R: 5'-GGCACAAAGCGACTGGATG-3'	XM_009448539.1<br>731-897	60	167	34
10	*Casepase9*	F: 5'-CCCTGGACGACATCTTTGA-3'<br>R: 5'-TAAAGCAACCAGGCATCTGT-3'	XM_009448829.1<br>1286-1402	60	117	34
11	*Beclin1*	F: 5'-GATGGAAGGGTCTAAGACGTCCAA-3'<br>R: 5'-TTTCGCCTGGGCTGTGGTAAG-3'	XM_010385905.1<br>145-304	60	160	34
12	*MMP9*	F: 5'-TGTACCGCTATGGTTACACT-3'<br>R:5'-CCTCAAAGGTTTGGAAT-3'	KJ897197.1<br>211-399	60	189	34
13	*VEGF*	F: 5'-GCGCTCGGTGCTGGAATTTG-3'<br>R:5'-TAGAGCAATCTCCCCAAGCCGTCG-3'	NM_001204384.1<br>118-276	60	159	34
14	*NF κ B*	F: 5'-GTGGACTACCTGGTGCCTCTA-3'<br>R: 5'-GTCCTTGGGTCCAGCAGTT-3'	KJ897243.1<br>268-461	60	194	34
15	*IL-1*	F: 5'- GACGCCCTCAATCAAAGT-3'<br>R:5'-CTTGGGCAGTCACATACA-3'	KJ891448.1<br>477-654	60	178	34
16	*IL-2*	F: 5'- GACTTTACTGCTGGATTT-3'<br>R: 5'- ATTGCTGATTAAGTCCCT-3'	NM_000586.3<br>160-379	60	220	34
17	*IL-4*	F: 5'- CAGTTCTACAGCCACCAT-3'<br>R: 5'- CTGGTTGGCTTCCTTCAC-3'	NM_172348.2<br>249-407	60	159	34
18	*IL-10*	F: 5'- TCAGGGTGGCGACTCTAT-3'<br>R:5'-TGGGCTTCTTTCTAAATCGTT-3'	NM_000572.2<br>601-799	60	199	34
19	*IL-17*	F: 5'- GAAGGCAGGAATCACAAT-3'<br>R:5'-ATCGGTTGTAGTAATCTG-3'	NM_002190.2<br>108-253	60	146	34
20	*FBXW7*	F: 5'-AGATGGACCAGGAGAGTG-3'<br>R:5'-CTTGCATGGTTTCTTTCC-3'	XM_009448414.1<br>554-771	60	218	34

21	*CD147*	F: 5'-TACTCCTGCGTCTTCCTCC-3'<br>R:5'-TGCGAGGAACTCACGAAG-3'	KJ896510.1<br>318-556	60	239	34
22	*GRP78*	F: 5'-GTTCTTGCCGTTCAAGGTGG-3'<br>R:5'-TGGTACAGTAACAACTGCATG-3'	FJ436356<br>600-780	60	181	34
23	*TOP1*	F: 5'-AAAGATCGAGAACACCGG-3'<br>R:5'-TGTTTGGTCTTCTCCTTCT-3'	XM_004062154.1<br>335-456	60	122	34
24	*TOP2*	F: 5'-AAAATGAAGATGCTAAGAAAAGACT-3'<br>R:5'-GTACAAACCAGGAACAAAAGTGACT-3'	XM_003315476.2<br>226-413	60	188	34
25	*GAPDH*	F: 5'-CAATGACCCCTTCATTGACC-3'<br>R: 5'- TGGAAGATGGTGATGGGATT-3'	NM_ 002046.3<br>201-335	60	135	34

Supplementary Table 2 The antibodies used in Western blot.

	Antibody	Species	Dilution	Company	Code number
1	BTG3	rabbit	1:500	sigma	HPA018400
2	p21 (F-5)	mouse	1:500	santa cruz	sc-6246
3	Cdk4 (C-22)	rabbit	1:500	santa cruz	sc-260
4	AIF (E-1)	mouse	1:700	santa cruz	sc-13116
5	LC3B	rabbit	1:1000	cell signaling	#2775
6	Beclin 1	rabbit	1:2000	abcam	Ab51031
7	β-catenin（C-18）	goat	1:500	santa cruz	sc-1496
8	p38α/β (H-147)	rabbit	1:500	santa cruz	sc-7149
9	β-actin(C4)	mouse	1:2000	santa cruz	sc-47778
